# Cold Treatment of Pneumonia

**Published:** 1891-01-17

**Authors:** 


### COLD TREATMENT OF PNEUMONIA.

We are still in doubt as to the part played by micro-organisms in the production of pneumonia. Koch, Friedlander, Klebs, and other observers have described specific micrococci, which have been found in the exudation, in the lymphatics of the lungs, and in blister fluid. Further, it has been stated that pneumonia has been produced in rabbits by the injection of cultivations of the pneumococcus, and in mice by the inhalation of a spray charged with these germs. The clinical histories of cases of pneumonia so forcibly confirm these views that the fact that similar micro-organisms have been found apart from pneumonia, can only lead us to suppose that we are unable to distinguish between micrococci, which are similar in appearance, but widely different in properties, or that in these latter cases the requisite conditions for the manifestation of their peculiar nature are wanting. Dr. Lipari, of Polerino, has demonstrated by his experiments the precise part played by cold in the production of pneumonia. He found that the simple endo-tracheal injection of the sputum of pneumonia, or the injection of the pleuritic exudation of animals which had died from pneumococci, did not produce pneumonia. If, however, the animal, either before the injection or soon after, was exposed to cold, the results were very different. Six out of eight of the animals treated in this way died with pneumonia. The alveoli were found to be infiltrated with exudation as in ordinary lobar pneumonia. Dr. Lipari is of opinion that the cold acts by paralysing the ciliated epithelium of the bronchi, causing their mucous membrane to swell at the same time. These two conditions would favour the entrance and descent of infectious matter into the alveoli. It is very possible, however, that the pneumococcus requires a low temperature for the development of its specific infective qualities. These researches have a practical bearing on the treatment of pneumonia by ice-bags and cold baths that have been re-introduced recently, a practice adopted in the seventeenth and eighteenth centuries, and more recently by Niemeyer twenty years ago. Dr. Lees has used the ice-bag in pneumonia during the last four years in eighteen cases, without a death, of these, two at least, in his opinion, would have certainly died without the ice-bag, and in two others of these cases would have almost certainly done so. By its use the temperature was lowered in all cases, but he found that the improvement did not consist only on the reduction of temperature. The physical signs were arrested in development, even before this crisis, and rapidly improved subsequently. He has never had any bad results with its use. Dr. Goodhart, too, has had excellent results with the ice-bag. In three out of his eighteen cases, symptoms of collapse ensued, but this was of a temporary nature and easily counteracted by the application of warmth to the feet and the administration of brandy. He warns us against adopting this method in children under two years of age. In hyperpyrexial cases the use of cold baths may save the patient when all other means fail. Dr. Angel Money has advocated the more general use of ice-bags in his treatment of broncho-pneumonia in children. He has treated many severe cases in this way with success, no matter what the cause of the disease was. The smaller the child the more marked the effects. He has used Leiter's tubes with advantage. The treatment must be carried out systematically in order to obtain any good results. He has found that the duration of the disease is on the whole shortened, and convalescence is almost invariably more rapid. Vomiting is staved off and diarrhœa not increased by the cold in cases in which these complications occur. Albuminuria is not rendered worse nor is otitis media more frequent than in cases treated without cold.

